# Trapping mammalian protein complexes in viral particles

**DOI:** 10.1038/ncomms11416

**Published:** 2016-04-28

**Authors:** Sven Eyckerman, Kevin Titeca, Emmy Van Quickelberghe, Eva Cloots, Annick Verhee, Noortje Samyn, Leentje De Ceuninck, Evy Timmerman, Delphine De Sutter, Sam Lievens, Serge Van Calenbergh, Kris Gevaert, Jan Tavernier

**Affiliations:** 1 VIB Medical Biotechnology Center, VIB, Ghent University, A. Baertsoenkaai 3, Ghent B-9000, Belgium; 2 Department of Biochemistry, Ghent University, A. Baertsoenkaai 3, Ghent B-9000, Belgium; 3 Laboratory for Medicinal Chemistry, Faculty of Pharmaceutical Sciences, Ghent University, Harelbekestraat 72, Ghent B-9000, Belgium

## Abstract

Cell lysis is an inevitable step in classical mass spectrometry–based strategies to analyse protein complexes. Complementary lysis conditions, *in situ* cross-linking strategies and proximal labelling techniques are currently used to reduce lysis effects on the protein complex. We have developed Virotrap, a viral particle sorting approach that obviates the need for cell homogenization and preserves the protein complexes during purification. By fusing a bait protein to the HIV-1 GAG protein, we show that interaction partners become trapped within virus-like particles (VLPs) that bud from mammalian cells. Using an efficient VLP enrichment protocol, Virotrap allows the detection of known binary interactions and MS-based identification of novel protein partners as well. In addition, we show the identification of stimulus-dependent interactions and demonstrate trapping of protein partners for small molecules. Virotrap constitutes an elegant complementary approach to the arsenal of methods to study protein complexes.

## Abstract (web summary)

A large portion of the proteome carries out its cellular function as part of macromolecular complexes. Here the authors describe Virotrap, a novel lysis-free approach for the isolation and identification of biologically relevant protein-protein and small molecule-protein interactions.

## Graphical abstract

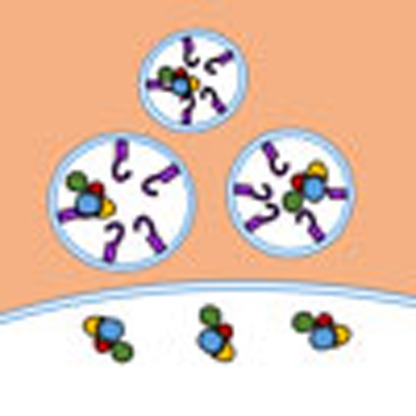

Proteins mostly exert their function within supramolecular complexes. Strategies for detecting protein–protein interactions (PPIs) can be roughly divided into genetic systems^1^ and co-purification strategies combined with mass spectrometry (MS) analysis (for example, AP–MS)^2^. The latter approaches typically require cell or tissue homogenization using detergents, followed by capture of the protein complex using affinity tags^3^ or specific antibodies^4^. The protein complexes extracted from this ‘soup' of constituents are then subjected to several washing steps before actual analysis by trypsin digestion and liquid chromatography–MS/MS analysis. Such lysis and purification protocols are typically empirical and have mostly been optimized using model interactions in single labs. In fact, lysis conditions can profoundly affect the number of both specific and nonspecific proteins that are identified in a typical AP–MS set-up. Indeed, recent studies using the nuclear pore complex as a model protein complex describe optimization of purifications for the different proteins in the complex by examining 96 different conditions^5^. Nevertheless, for new purifications, it remains hard to correctly estimate the loss of factors in a standard AP–MS experiment due to washing and dilution effects during treatments (that is, false negatives). These considerations have pushed the concept of stabilizing PPIs before the actual homogenization step. A classical approach involves cross-linking with simple reagents (for example, formaldehyde) or with more advanced isotope-labelled cross-linkers (reviewed in ref. 2). However, experimental challenges such as cell permeability and reactivity still preclude the widespread use of cross-linking agents. Moreover, MS-generated spectra of cross-linked peptides are notoriously difficult to identify correctly. A recent lysis-independent solution involves the expression of a bait protein fused to a promiscuous biotin ligase, which results in labelling of proteins proximal to the activity of the enzyme-tagged bait protein^6^. When compared with AP–MS, this BioID approach delivers a complementary set of candidate proteins, including novel interaction partners^7^,^8^. Such particular studies clearly underscore the need for complementary approaches in the co-complex strategies.

The evolutionary stress on viruses promoted highly condensed coding of information and maximal functionality for small genomes. Accordingly, for HIV-1 it is sufficient to express a single protein, the p55 GAG protein, for efficient production of virus-like particles (VLPs) from cells^9^,^10^. This protein is highly mobile before its accumulation in cholesterol-rich regions of the membrane, where multimerization initiates the budding process^11^. A total of 4,000–5,000 GAG molecules is required to form a single particle of about 145 nm (ref. 12). Both VLPs and mature viruses contain a number of host proteins that are recruited by binding to viral proteins. These proteins can either contribute to the infectivity (for example, Cyclophilin/FKBPA^13^) or act as antiviral proteins preventing the spreading of the virus (for example, APOBEC proteins^14^).

We here describe the development and application of Virotrap, an elegant co-purification strategy based on the trapping of a bait protein together with its associated protein partners in VLPs that are budded from the cell. After enrichment, these particles can be analysed by targeted (for example, western blotting) or unbiased approaches (MS-based proteomics). Virotrap allows detection of known binary PPIs, analysis of protein complexes and their dynamics, and readily detects protein binders for small molecules.

## Results

### Concept of the Virotrap system

**Figure 1:**
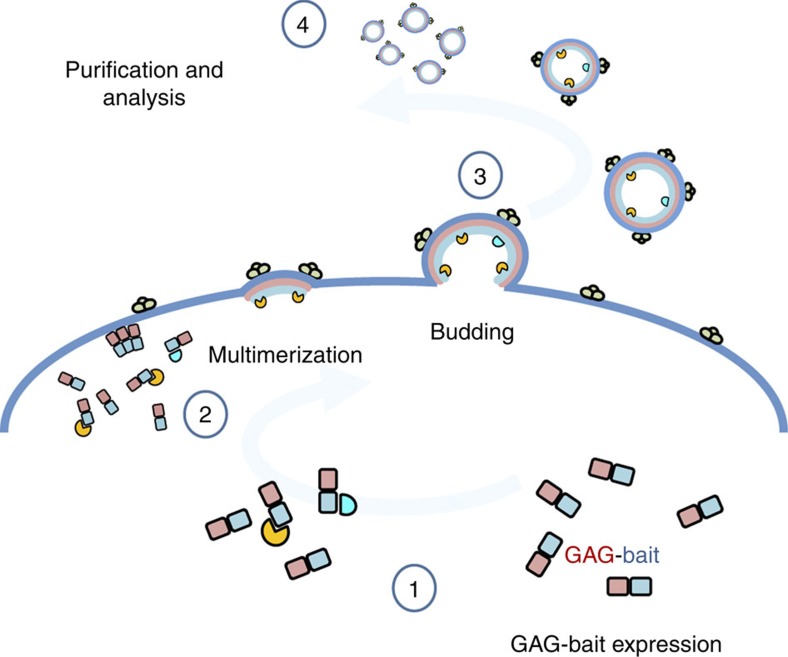
Schematic representation of the Virotrap strategy. Expression of a GAG-bait fusion protein (1) results in submembrane multimerization (2) and subsequent budding of VLPs from cells (3). Interaction partners of the bait protein are also trapped within these VLPs and can be identified after purification by western blotting or MS analysis (4).

Classical AP–MS approaches rely on cell homogenization to access protein complexes, a step that can vary significantly with the lysis conditions (detergents, salt concentrations, pH conditions and so on)^5^. To eliminate the homogenization step in AP–MS, we reasoned that incorporation of a protein complex inside a secreted VLP traps the interaction partners under native conditions and protects them during further purification. We thus explored the possibility of protein complex packaging by the expression of GAG-bait protein chimeras (Fig. 1) as expression of GAG results in the release of VLPs from the cells^9^,^10^. As a first PPI pair to evaluate this concept, we selected the HRAS protein as a bait combined with the RAF1 prey protein. We were able to specifically detect the HRAS–RAF1 interaction following enrichment of VLPs via ultracentrifugation (Supplementary Fig. 1a). To prevent tedious ultracentrifugation steps, we designed a novel single-step protocol wherein we co-express the vesicular stomatitis virus glycoprotein (VSV-G) together with a tagged version of this glycoprotein in addition to the GAG bait and prey. Both tagged and untagged VSV-G proteins are probably presented as trimers on the surface of the VLPs, allowing efficient antibody-based recovery from large volumes. The HRAS–RAF1 interaction was confirmed using this single-step protocol (Supplementary Fig. 1b). No associations with unrelated bait or prey proteins were observed for both protocols.

### Virotrap for the detection of binary interactions

**Figure 2:**
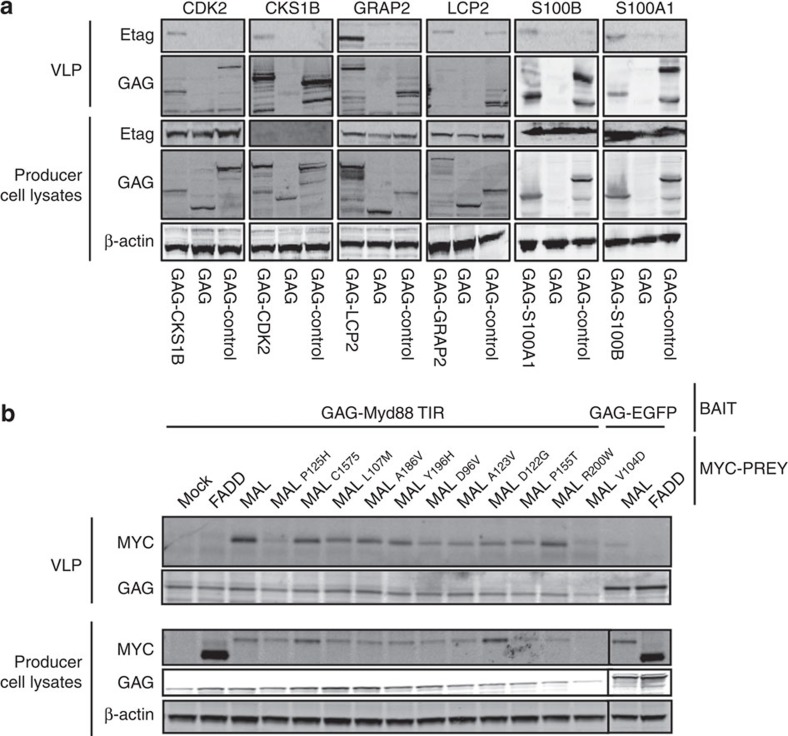
Virotrap experiments for binary PPI detection. (**a**) Virotrap experiments for reciprocal detection of binary protein–protein interactions. HEK293T cells were transfected with GAG-bait constructs and E-tagged prey constructs. Additional co-transfection of VSV-G/FLAG-VSV-G expression constructs allowed efficient purification, which was followed by direct on-bead lysis and analysis by western blotting using anti-E-tag (for the presence of the prey protein), anti-GAG (bait expression levels and particles) and anti-β-actin antibodies. A representative experiment is shown for three biological repeat experiments. (**b**) Virotrap analysis of weak protein–protein interactions. Cells were transfected with MYD88 TIR bait constructs or control bait plasmids (GAG-EGFP) and combined with MYC-tagged MAL prey constructs or FADD prey controls. In the western blot analysis, proteins were revealed using anti-MYC for prey presence, anti-GAG for bait expression and VLP formation, and anti-β-actin for normalization of lysates. Analyses were performed for VLPs and for lysates of the producer cells. A representative experiment for three biological experiments is shown. Uncropped gel images and molecular weight markers are presented in Supplementary Fig. 10.

We next explored the reciprocal detection of a set of PPI pairs, which were selected based on published evidence and cytosolic localization^15^. After single-step purification and western blot analysis, we could readily detect reciprocal interactions between CDK2 and CKS1B, LCP2 and GRAP2, and S100A1 and S100B (Fig. 2a). Only for the LCP2 prey we observed nonspecific association with an irrelevant bait construct. However, the particle levels of the GRAP2 bait were substantially lower as compared with those of the GAG control construct (GAG protein levels in VLPs; Fig. 2a, second panel of the LCP2 prey). After quantification of the intensities of bait and prey proteins and normalization of prey levels using bait levels, we observed a strong enrichment for the GAG-GRAP2 bait (Supplementary Fig. 2).

To compare Virotrap with other technologies, we performed a binary analysis of the human positive reference set (hsPRS-v1, 92 PPI pairs) and the corresponding random reference set (hsRRS-v1, 92 randomly selected pairs). Both sets contain proteins from all cellular compartments to remove any bias in protein localization. In this western blotting screen, all 184 baits and 184 preys were expressed in combinations as published by Braun *et al*.^15^. After expression, purification and western blot analysis, we were able to detect 28 (30%) interactions in the PRS, whereas 5 (5%) interactions were detected in the RRS (Supplementary Data 1 and Supplementary Fig. 3). Western blot analysis of the lysates of the producer cells showed that ∼30% of the bait proteins (56 out of 184) and 40% of the prey proteins (E-tag, 25 out of 41) were not expressed at a detectable level, indicating a large underestimation of detectable interactions (Supplementary Data 1). Supplementary Fig. 3 shows the overlap between Virotrap and data obtained with other PPI methods for the PRS as published by Braun *et al*.^15^, underscoring the notion that Virotrap detects known interactions and further confirms additional PPIs from the PRS, complementary to the other approaches.

To further evaluate the sensitivity of Virotrap, we assessed the interaction between two signalling molecules in Toll-like receptor signalling: myeloid differentiation primary response 88 (MYD88) and MAL (TIRAP), which bind via their Toll/interleukin1 receptor (TIR) homology domains^16^ (*K*_d_=8 μM (ref. 17)). A panel of MYC-tagged MAL mutant prey proteins with reduced binding affinities^18^ was tested against the MYD88 TIR domain as bait. Figure 2b shows western blotting results for prey presence in the particles. These binary Virotrap experiments generally show the same trend as the data obtained with the mammalian PPI trap (MAPPIT) assay^18^(Supplementary Fig. 4), a mammalian two-hybrid method for which the readout reflects the affinity between protein partners^19^. In addition, these data illustrate that Virotrap enables the detection of weak PPIs.

### Virotrap for unbiased discovery of novel interactions

**Figure 3:**
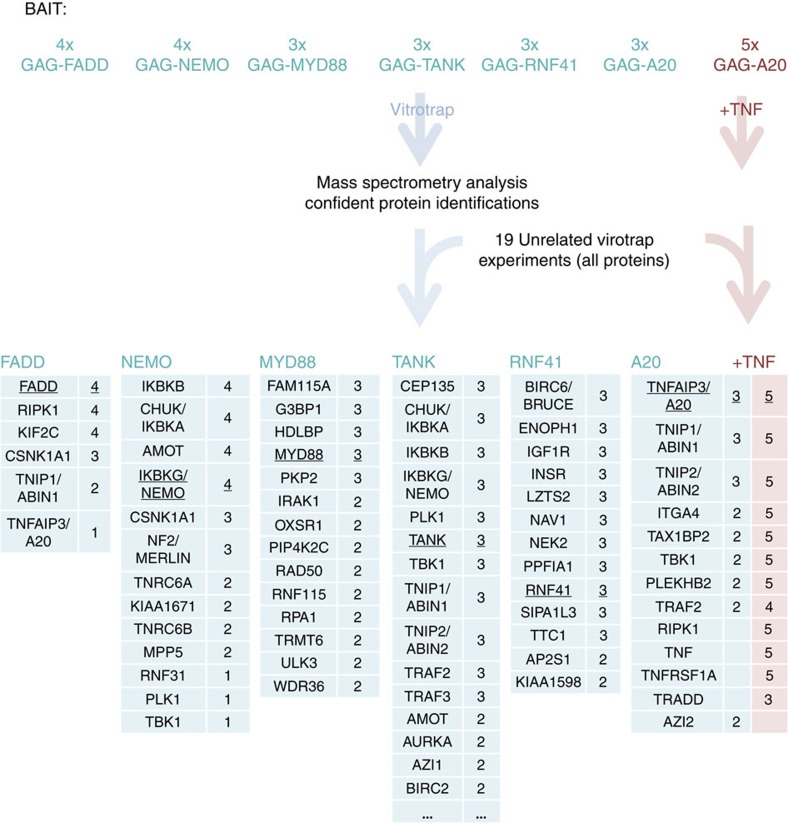
Use of Virotrap for unbiased interactome analysis. A total of three (A20, TANK, MYD88 and RNF41) or four (FADD and NEMO) transfections were performed for interactome profiling. After single-step purification, specific elution, lysis and protein digestion, samples were analysed by liquid chromatography–tandem mass spectrometry. The obtained data were challenged with all the identifications obtained for 19 unrelated Virotrap experiments. The tables show the candidate interaction partners for the different baits identified with at least two peptides. The number of protein identifications in the biological repeats for the different baits is shown next to the gene name identifier. Higher recurrence is expected to increase confidence. Proteins in bold were described before (BioGRID^3.2^). Analysis of the A20 interactome after activation of the TNF pathway is shown as one of the conditions (in red font). Five transfections were performed for this condition.

For the detection of novel interaction partners, we scaled up VLP production and purification protocols (Supplementary Fig. 5 and Supplementary Note 1 for an overview of the protocol) and investigated protein partners trapped using the following bait proteins: Fas-associated via death domain (FADD), A20 (TNFAIP3), nuclear factor-κB (NF-κB) essential modifier (IKBKG), TRAF family member-associated NF-κB activator (TANK), MYD88 and ring finger protein 41 (RNF41). To obtain specific interactors from the lists of identified proteins, we challenged the data with a combined protein list of 19 unrelated Virotrap experiments (Supplementary Table 1 for an overview). Figure 3 shows the design and the list of candidate interactors obtained after removal of all proteins that were found in the 19 control samples (including removal of proteins from the control list identified with a single peptide). The remaining list of confident protein identifications (identified with at least two peptides in at least two biological repeats) reveals both known and novel candidate interaction partners. All candidate interactors including single peptide protein identifications are given in Supplementary Data 2 and also include recurrent protein identifications of known interactors based on a single peptide; for example, CASP8 for FADD and TANK for NEMO. Using alternative methods, we confirmed the interaction between A20 and FADD, and the associations with transmembrane proteins (insulin receptor and insulin-like growth factor receptor 1) that were captured using RNF41 as a bait (Supplementary Fig. 6). To address the use of Virotrap for the detection of dynamic interactions, we activated the NF-κB pathway via the tumour necrosis factor (TNF) receptor (TNFRSF1A) using TNFα (TNF) and performed Virotrap analysis using A20 as bait (Fig. 3). This resulted in the additional enrichment of receptor-interacting kinase (RIPK1), TNFR1-associated via death domain (TRADD), TNFRSF1A and TNF itself, confirming the expected activated complex^20^.

Further, Virotrap was compared side-by-side with classical AP–MS experiments using A20 and RNF41 as baits. Both overlapping and unique prey proteins were identified for these bait proteins (Supplementary Fig. 7). Taken together, when compared with classical AP–MS, Virotrap provides a complementary view on bait interactomes.

**Figure 4:**
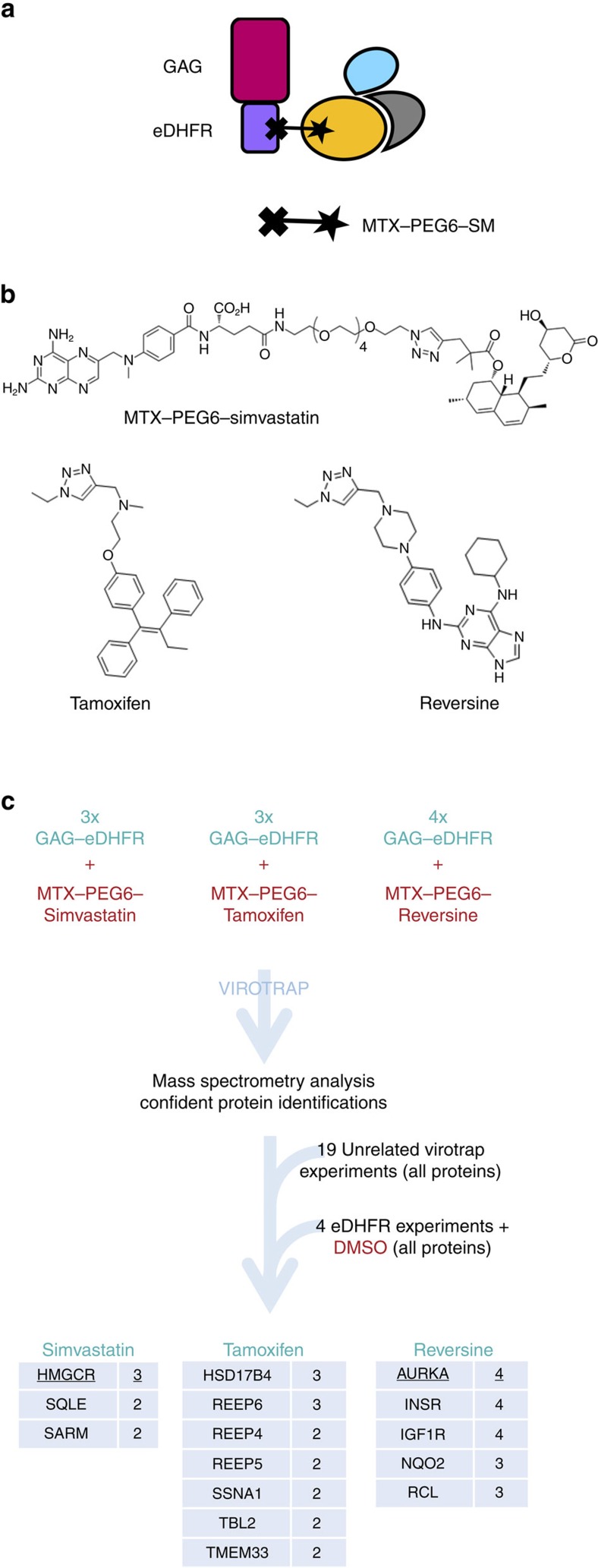
Use of Virotrap for detection of protein partners of small molecules. (**a**) Scheme for the application of Virotrap for small molecules. *E. coli* DHFR is coupled to GAG, allowing the direct recruitment of methotrexate (MTX) fused via a polyethylene glycol linker (PEG6) to a small molecule of interest (SM). (**b**) Chemical structure of the bivalent molecules used in this study. For tamoxifen and reversine, only the active compound with the linker group is shown. (**c**) Design of the Virotrap study for proteins binding to simvastatin (three replicates), tamoxifen (three replicates) and reversine (four replicates). Control samples consisted of 19 experiments with unrelated bait proteins (see higher) and 4 DMSO-treated GAG-eDHFR Virotrap experiments. The high confidence results (more than one peptide in two biological replicates) are shown in the specific tables.

We additionally explored the use of Virotrap for the detection of protein interactions with small molecules. To this end, we fused the *Escherichia coli* dihydrofolate reductase protein (eDHFR) as a bait to GAG and treated the particle-producing cells with bivalent molecules consisting of methotrexate (MTX) linked via a polyethylene glycol linker to a small molecule of interest (Fig. 4a). In this study, we used simvastatin, tamoxifen and reversine as small molecule baits (Fig. 4b (ref. 21)). Data analysis was performed by elimination of all proteins previously identified in the 19 control experiments (as before) combined with the protein identifications from 4 additional eDHFR bait experiments treated with dimethyl sulfoxide (DMSO). This resulted in the consistent enrichment of the known targets for simvastatin (HMG-CoA reductase enzyme (HMGCR)) and reversine (Aurora kinase A (AURKA)). The novel interactions of tamoxifen with HSDB17B4, and reversine with NQO2 were confirmed using an orthogonal MASPIT assay^22^ (Supplementary Fig. 8).

An important issue in AP–MS is defining the actual set of background, nonspecific interactors^23^,^24^. Analysis of 19 Virotrap control samples reveals a high recurrence of ∼174 proteins (identified in at least 15/19 samples) (Supplementary Note 2 and Supplementary Fig. 9). Structural proteins (for example, ACTB and EZR), serum proteins (for example, A2M and albumin) and proteins related to HIV biology (GAG and its host interaction partners such as Cyclophilin/PPIA and ALIX/PDCD6IP) are found as highly recurrent background proteins. Further, the distribution of the abundance of these background proteins is similar for Virotrap and AP–MS, although there is a high recurrence in the background of known low abundant proteins, which is likely to be due to the unique biology underlying Virotrap (Supplementary Fig. 9).

## Discussion

Lysis conditions used in AP–MS strategies are critical for the preservation of protein complexes. A multitude of lysis conditions have been described, culminating in a recent report where protein complex stability was assessed under 96 lysis/purification protocols^5^. Moreover, the authors suggest to optimize the conditions for every complex, implying an important workload for researchers embarking on protein complex analysis using classical AP–MS. As lysis results in a profound change of the subcellular context and significantly alters the concentration of proteins, loss of complex integrity during a classical AP–MS protocol can be expected. A clear evolution towards ‘lysis-independent' approaches in the co-complex analysis field is evident with the introduction of BioID^6^ and APEX^25^ where proximal proteins, including proteins residing in the complex, are labelled with biotin by an enzymatic activity fused to a bait protein. A side-by-side comparison between classical AP–MS and BioID showed overlapping and unique candidate binding proteins for both approaches^7^,^8^, supporting the notion that complementary methods are needed to provide a comprehensive view on protein complexes. This has also been clearly demonstrated for binary approaches^15^ and is a logical consequence of the heterogenic nature underlying PPIs (binding mechanism, requirement for posttranslational modifications, location, affinity and so on).

In this report, we explore an alternative, yet complementary method to isolate protein complexes without interfering with cellular integrity. By trapping protein complexes in the protective environment of a virus-like shell, the intact complexes are preserved during the purification process. This constitutes a new concept in co-complex analysis wherein complex stability is physically guaranteed by a protective, physical structure. A comparison of our Virotrap approach with AP–MS shows complementary data, with specific false positives and false negatives for both methods (Supplementary Fig. 7).

The current implementation of the Virotrap platform implies the use of a GAG-bait construct resulting in considerable expression of the bait protein. Different strategies are currently pursued to reduce bait expression including co-expression of a native GAG protein together with the GAG-bait protein, not only reducing bait expression but also creating more ‘space' in the particles potentially accommodating larger bait protein complexes. Nevertheless, the presence of the bait on the forming GAG scaffold creates an intracellular affinity matrix (comparable to the early *in vitro* affinity columns for purification of interaction partners from lysates^26^) that has the potential to compete with endogenous complexes by avidity effects. This avidity effect is a powerful mechanism that aids in the recruitment of cyclophilin to GAG^27^, a well-known weak interaction (*K*_d_=16 μM (ref. 28)) detectable as a background association in the Virotrap system. Although background binding may be increased by elevated bait expression, weaker associations are readily detectable (for example, MAL—MYD88-binding study; Fig. 2c).

The size of Virotrap particles (around 145 nm) suggests limitations in the size of the protein complex that can be accommodated in the particles. Further experimentation is required to define the maximum size of proteins or the number of protein complexes that can be trapped inside the particles.

The underlying biology of the system makes it best suited for the study of cytosolic complexes. However, we could identify prey proteins residing in the plasma membrane compartment or in the endoplasmic reticulum (ER) membrane. The current version of the platform is probably not applicable to mitochondria, peroxisomes or other confined cellular compartments. The introduction of alternative or artificial viral matrix variants combined with adapted purification protocols will open the way to protein complex trapping in other compartments of the cell. The activity of antiviral factors (for example, tetherin^29^) may interfere with Virotrap in other cell types. Via genome engineering approaches, these factors can be eliminated. The use of artificial or mutated GAG variants can also prevent the activity of antiviral mechanisms and may thus facilitate the introduction of the system in other cell lines and primary cells.

It is still highly challenging to find protein interaction partners for small molecules, not only for clarifying the mechanism of action of orphan drugs but also for identification of the proteins responsible for the off-target effects of known drugs. Besides the classical pull-down approach using biotinylated variants of the small molecules, some technologies were specifically developed for this purpose including MAPPIT variants^22^,^30^ and the more recent thermal profiling strategy coupled to proteomics^31^. Using a bivalent small molecule as a bait we here show successful capture of the well-known simvastatin target HMGCR, a transmembrane ER-resident protein. UBIAD1 (ref. 32) is probably a member of the HMGCR complex that is co-sorted to the particles. A physical interaction between HMGCR and SQLE has not been formally shown, but the specific identification by Virotrap of this second rate-limiting enzyme in the cholesterol pathway may point to a beneficial off-target effect of simvastatin.

In this study we employ a stringent filtering approach to reveal specific protein partner candidates based on the elimination of all proteins identified in a large number of control experiments (19 controls for standard discovery+4 additional DMSO experiments for the small molecule application). A number of filtering approaches have been described^24^ and the careful application of some of these tools may provide a useful alternative for the current ‘black list' strategy. For example, application of the bait-centred tool SFINX^33^ using AURKA as artificial bait for the reversine study revealed association with TTK (SFINX P value of 1.79E−43), a well-known target of this small molecule^34^. However, TTK is removed in the black list strategy, because of sporadic identification in control experiments.

In conclusion, Virotrap captures significant parts of known interactomes and reveals new interactions. This cell lysis-free approach purifies protein complexes under native conditions and thus provides a powerful method to complement AP–MS or other PPI data. Future improvements of the system include strategies to reduce bait expression to more physiological levels and application of advanced data analysis options to filter out background. These developments can further aid in the deployment of Virotrap as a powerful extension of the current co-complex technology arsenal.

## Methods

### Plasmids and antibodies

The p55 GAG fusion constructs were generated by PCR amplification of the p55 GAG coding sequence from the pCMV-dR8.74 packaging construct (Addgene) and by subsequent In-Fusion reaction (Clontech) in pMG1-Ras, a HRAS expression vector used in the MAPPIT system^35^, resulting in a p55 GAG-RAS under control of the strong SRalpha promoter (pMET7-GAG-Ras). Enhanced green fluorescent protein (EGFP) was transferred from pEGFP-C1 vector (Clontech) to generate the pMET7-GAG-EGFP construct. Using PCR-based cloning, a Gateway cassette was inserted to allow recombination-assisted cloning. The complete set of positive and random reference clones were transferred in a single direction (no bait–prey swap) using standard GATEWAY cloning. Prey open reading frames from these sets were transferred into a GATEWAY-compatible pMET7 expression vector with an amino-terminal E-tag fused in frame. The MYD88 TIR and MAL prey constructs were generated from plasmids that were previously described^18^. The complementary DNAs for A20, RNF41, TANK, FADD and eDHFR were transferred from the ORFEOME 5.1 collection or from previous constructs^22^,^36^ into the pMET7 vector containing in-frame gateway sites and N-terminal epitope tags (MYC-, FLAG- or VSV) or an N-terminal GAG sequence, to allow Virotrap experiments. The TNFR plasmid (pSV25S-hTNFR 55) was obtained from the BCCM/LMBP plasmid collection.

The pMD2.g pseudotyping vector was kindly provided by D. Trono (EPFL, Lausanne, Switzerland; Addgene). The pcDNA3-FLAG-VSV-G and pcDNA3-Etag-VSV-G will be described elsewhere.

Antibodies used for western blotting were anti-p24 GAG (Abcam; ab9071; 1/1,000), anti-FLAG (M2, Sigma Aldrich; 1/2,000), anti-actin (Sigma Aldrich; A2066; 1/2,000), anti-VSV (Sigma Aldrich; V4888; 1/1,000), anti-MYC (clone 4E10, prepared in-house) and anti-E-tag (GE Healthcare; 27941201; 1/1,000). Secondary antibodies were from Li-Cor (1/5,000) and blottings were digitally imaged using an ODYSSEY Imager system (Li-Cor).

### Production and purification of Virotrap particles

Standard HEK293T cells (obtained from the Rufer lab at the CHUV, Lausanne) were cultured in a humidified atmosphere at 8% CO_2_ using high-glucose DMEM (Invitrogen) complemented with 10% FCS and antibiotics. Cell cultures were kept at low passage (<10) and regularly tested for mycoplasma contamination.

Cells were transfected overnight the day after seeding, with a standard calcium phosphate transfection procedure. For ultracentrifugation experiments, we transfected 25 μg of bait vector (GAG-EGFP and GAG-HRAS) normalized to 50 μg with a mock vector, in 6 × 10^6^ cells seeded the day before in 75 cm^2^ bottles. For concentration of the VLPs, we harvested supernatant after 24 h, centrifuged samples for 3 min at 140 *g* to remove cellular debris and filtered the supernatant through 0.45 μm filters. The samples were then centrifuged in a Beckman ultracentrifuge using a Ti41 swinging bucket rotor at 22,000 r.p.m. The supernatant was discarded and VLP pellets were re-suspended directly in loading buffer for western blot analysis.

For binary interaction assays, 650,000 HEK293T cells were seeded the day before transfection in 6-well plates. On the day of transfection, a DNA mixture was prepared containing the following: 3.5 μg bait construct (pMET7-GAG-bait), 0.8 μg prey construct (pMET7-E-tag prey or pMET7-FLAG-RAF1), 0.7 μg pMD2.G and 1.4 μg pcDNA3-FLAG-VSV-G. Following overnight transfection, cells were washed once with PBS and 1 ml of fresh growth medium was added to the wells. Cellular debris was removed from the harvested supernatant by 3 min centrifugation at 400 *g*. The cleared medium was then incubated with 10 μl Dynabeads MyOne Streptavidin T1 beads (Invitrogen) pre-loaded with 1 μg monoclonal ANTI-FLAG BioM2-Biotin, Clone M2 (Sigma-Aldrich) according to the manufacturer's protocol. After 2 h binding at 4 °C by end-over-end rotation, beads were washed two times with washing buffer (20 mM HEPES pH 7.4 and 150 mM NaCl) and the captured particles were released directly in 35 μl 2 × SDS–PAGE loading buffer. A 5-min incubation step at 65 °C before removal of the beads ensured complete release. After boiling, the samples were loaded on a 10% SDS–PAGE gel or on commercial 4–12% gradient gels (Biorad) and after separation the proteins were transferred to Hybond-C Extra nitrocellulose membranes (GE Healthcare). Lysates of the producer cells were prepared by direct addition of 200 μl RIPA buffer (50 mM Tris-HCl pH 7.4, 150 mM NaCl, 1% NP40, 1% sodium deoxycholate, 0.1% SDS and Complete protease inhibitor cocktail (Roche)) to the 6-well plates after washing of the cells in chilled PBS. The lysates were cleared by centrifugation at 13,000 *g*, 4 °C for 15 min, to remove the insoluble fraction.

The PRS and RRS were randomized and processed in sets of about 45 experiments. Each set was loaded on 2 4–12% gradient gels with 26 slots (Biorad). Each set of experiments also contained the GAG-EGFP expression control, a mock control and the interaction between GRAP2 and LCP2 as a positive control for Virotrap functionality. A single pooled positive control for the GRAP2-LCP2 interaction was also loaded on each gel to allow cross-comparison between the gels. Bands were quantified by fluorescence signals with an ODYSSEY system (Li-Cor). The detection threshold was based on RRS signals and was set at 28 positive signals from the positive reference set, at the expense of 5 false-positive measures from the random reference set.

For MS, 10^7^ HEK293T cells were seeded in three to four 75 cm^2^ bottles and transfected the next day with a total of 15 μg DNA per bottle using polyethyleneimine (PEI) reagent. The following DNA/PEI quantities were used: GAG-bait 7.5 μg; mock vector 5.4 μg; 2.1 μg of a 1/2 pMD2.G-pcDNA3-FLAG-VSV-G mix versus 37.5 μl PEI. The cellular supernatant was harvested after 32 h and centrifuged for 3 min at 450 *g*, to remove cellular debris. For simvastatin, tamoxifen and reversine experiments, the bivalent MTX-polyethylene glycol-small molecules^21^,^37^ were added after transfection to the producing cells at a concentration of 1 μM. Producer cells transfected with the A20 bait, VSV-G capture proteins and 1.15 μg TNFR plasmid were treated with 300 IU ml^−1^ of human TNFα during production, to monitor dynamic A20 complexes. The cleared supernatant was then filtered using 0.45 μm filters (Millipore). A total of 100 μl MyOne Streptavidin T1 beads pre-loaded with 10 μl ANTI-FLAG BioM2-Biotin antibody was used to bind the tagged particles. Particles were allowed to bind for 2 h by end-over-end rotation. The total supernatant was processed in three consecutive binding steps. Bead-particle complexes were washed once with washing buffer (20 mM Tris-HCl pH 7.5 and 150 mM NaCl) and were then eluted with FLAG peptide (30 min at 37 °C; 200 μg ml^−1^ in washing buffer) and lysed by addition of SDS to a final concentration of 0.1%. After 5 min, SDS was removed using HiPPR Detergent Removal Spin Columns (Pierce, Thermo Scientific) followed by boiling and overnight digestion with 0.5 μg sequence-grade trypsin (Promega). After acidification (addition of 1 μl of 10% trifluoroacetic acid), the peptides were separated by nano-LC and directly analysed with a Q Exactive instrument (Thermo Scientific) operating in MS/MS mode as described before^38^. Searches were performed using the MASCOT algorithm (Version 2.4.1, Matrix Science) at 99% confidence against human and bovine SWISSPROT accessions (Release 2013_02) complemented with HIV-1, EGFP, VSV-G and FLAG-VSV-G protein sequences. False discovery rates (FDR) were obtained by searches against the reversed version of the complete search database and by retaining only the peptide to spectra matches (PSMs) with the highest score in standard or reverse search. The false discovery rate was then calculated by dividing the number of PSMs against the reversed database by the number of PSMs against both databases. Identifications are reported by unique gene name in the tables. Raw data files, search settings, mascot generic format files (MGFs) and identification lists were submitted to PRIDE using Proteome Exchange. Protein spectral count files and peptide spectral count files are provided as Excel files (Supplementary Data 3 and 4, respectively).

### AP–MS experiments

Plasmids encoding N-terminally FLAG-tagged A20 and RNF41 were transfected using PEI in 2 × 15-cm dishes. Cells were scraped in lysis buffer (50 mM HEPES–KOH pH 8.0, 100 mM KCl, 2 mM EDTA, 0.1% NP40, 10% glycerol, 1 mM dithiothreitol, 0.5 mM phenylmethylsulfonyl fluoride, Protease inhibitor cocktail (Roche), 0.25 mM sodium orthovanadate, 50 mM glycerophosphate and 10 mM NaF) and then processed according to the protocol described by Kean *et al*.^39^. MS analysis was performed as described higher for Virotrap samples. MASCOT searches were performed at 99% confidence against the human SWISSPROT database. Supplementary Table 2 shows an overview of the obtained results. Protein spectral count files and peptide spectral count files are also provided as Excel files (Supplementary Data 5 and 6, respectively).

### Co-immunoprecipitation and MAPPIT/MASPIT experiments

For co-immunoprecipitation experiments, 4–4.5 × 10^6^ HEK293T were seeded in 10 cm dishes and transfected the next day with 17 μg of each tagged protein. Cells were lysed in lysis buffer (10 mM Tris-HCl pH 8, 150 mM NaCl, 1% NP40, 10% glycerol, 5 μM ZnCl_2_, phosphatase inhibitors and protease inhibitor cocktail (Complete; Roche)). Immunoprecipitation was performed with 10 μl MyOne Streptavidin T1 beads preloaded with 1 μg ANTI-FLAG BioM2-Biotin antibody. Control immunoprecipitations were performed without ANTI-FLAG antibody. The material was directly eluted using SDS–PAGE loading buffer, boiled and loaded on SDS–PAGE. MAPPIT experiments were performed as described before^36^. The binary MASPIT assays were essentially performed as described before^21^. Briefly, HEK293T cells were seeded in black, tissue-culture-treated 96-well plates at 10,000 cells per well in 100 μl culture medium (DMEM supplemented with 10% FCS) and grown at 37 °C, 8% CO_2_. Twenty-four hours later, cells were transfected with a combination of the pCLG-eDHFR bait plasmid^21^, the pMG1-HSD17B4 prey construct and the pXP2d2-rPAP1-luciferase reporter^22^. The prey constructs were generated by Gateway transfer of the full-size AURKA, ESR1, HSD17B4 and NQO2 open reading frames, obtained as an entry clone in the hORFeome collection, into the Gateway compatible pMG1 prey destination vector^40^. Twenty-four hours after transfection, cells were either left unstimulated or treated with 100 ng ml^−1^ leptin, with addition of DMSO, MTX-modified tamoxifen or MTX-modified Reversine. Another 24 h later, luciferase activity was assayed using the Luciferase Assay System kit (Promega).

## Additional information

**Accession codes:** The mass spectrometry proteomics data have been deposited to the ProteomeXchange Consortium via the PRIDE partner repository^41^ with the data set identifier PXD000685 and 10.6019/PXD000685.

**How to cite this article:** Eyckerman, S. *et al*. Trapping mammalian protein complexes in viral particles. *Nat. Commun.* 7:11416 doi: 10.1038/ncomms11416 (2016).

## Supplementary Material

Supplementary Figures, Supplementary Tables, Supplementary Notes and Supplementary References Supplementary Figures 1-10, Supplementary Tables 1-2, Supplementary Notes 1-2 and Supplementary References

Supplementary Data 1 Results obtained with binary Virotrap for positive (PRS) and random reference sets (RRS).

Supplementary Data 2 All specific protein identifications for the different baits in this study.

Supplementary Data 3 Supplementary Table Spectral Counts file for Virotrap experiments

Supplementary Data 4 Peptide identifications for the Virotrap data.

Supplementary Data 5 Protein Spectral Counts for AP-MS data on RNF41, A20 and on EGFP controls

Supplementary Data 6 Peptide identifications for the AP-MS data on RNF41, A20 and EGFP controls.
