# Supplementary material for: Trapping mammalian protein complexes in viral particles
Source: Nat Commun. 2016 Apr 28;7:11416. doi: 10.1038/ncomms11416 (PMC4853472; doi:10.1038/ncomms11416)
Supplement: Supplementary Figures, Supplementary Tables, Supplementary Notes and Supplementary References — Supplementary Figures 1-10, Supplementary Tables 1-2, Supplementary Notes 1-2 and Supplementary References [file ncomms11416-s7.pdf]

Supplementary figures

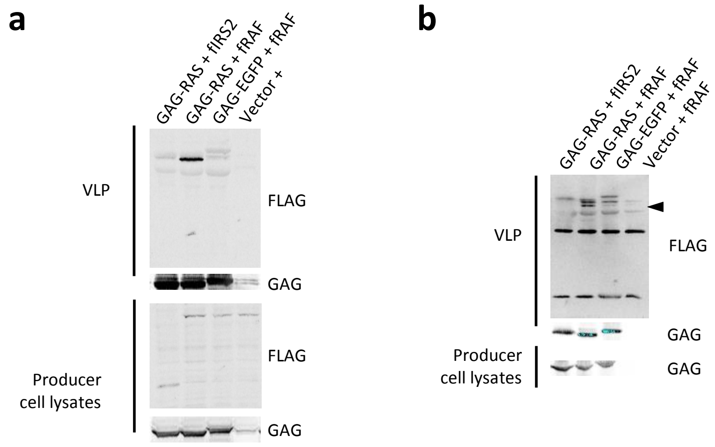

Supplementary Fig. 1: Binary Virotrap analysis of the HRAS – RAF1 interaction. a. Enrichment of particles by ultracentrifugation. Supernatants from HEK293T cells transfected with different combinations of bait proteins (GAG-EGFP (control) and GAG-RAS) and FLAG-tagged prey proteins (IRS2 and RAF), were harvested after 24 hours, and processed by ultracentrifugation to pellet the particles. Particle pellets were lysed in loading buffer, separated by SDS-PAGE and probed after Western blotting with anti-FLAG and anti-GAG antibodies. **b**: **Enrichment of particles by a single step VLP enrichment protocol.** HEK293T cells were seeded in 6 well plates and transfected with bait and prey combinations as in (a). Both wild-type and E-tagged VSV-G glycoproteins were expressed to allow particle enrichment via a single step protocol from a 1 ml harvest. Western blotting of the eluted particles and the lysates was performed with anti-FLAG and anti-GAG antibodies. The expression level of the prey proteins was below the detection limit in the lysates. All experiments were performed at least 3 times (biological repeats). The results of one representative experiment are shown. Uncropped gel images and molecular weight markers are presented in **Supplementary Fig. 10**.

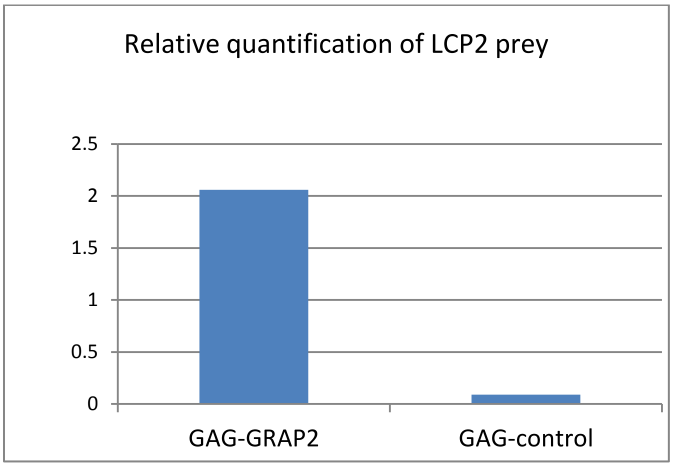

Supplementary Fig. 2: Detection of the LCP2 – GRAP2 interaction in Virotrap. Levels of GAG-GRAP2 and GAG-control bands were quantified using imaging software (Li-Cor, ODYSSEY®). The GAG-bait expression levels were then used as normalization for the LCP2 prey as detected by Western blot in the Virotrap particles. A 23-fold increase in relative abundance of the prey was detected when a relevant GAG-bait is present. This analysis was done on a representative experiment out of 3 biological repeats.

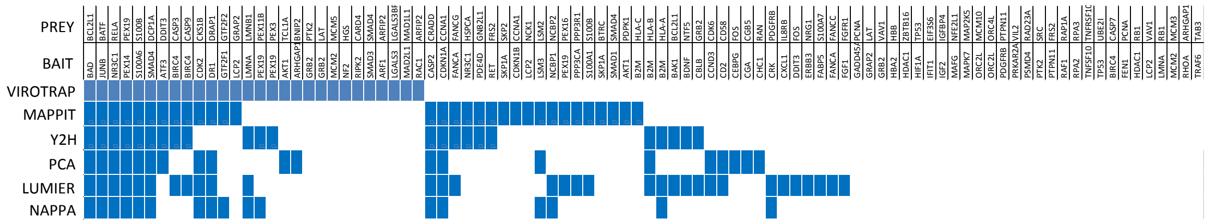

Supplementary Fig. 3: Binary Virotrap assays for the Positive Reference Set (PRS)[1] and comparison against other binary protein-protein interaction assays. All 184 interaction pairs from both the PRS and the Random Reference Set (RRS) were explored by a transfection experiment in HEK293T cells, followed by single step purification and Western blot analysis of the eluted particles and lysates of the producer cells. Prey protein presence was revealed by anti-E-tag antibodies on the VLP samples. The colored blocks show that Virotrap results in 30% positive interactions at the expense of 5% false positive signals in the RRS set (not shown, see **Supplementary Table 1**). For the other methods, we used data from Braun *et al.*, 2009 [1]. This full PRS and RRS western screening analysis was performed once.

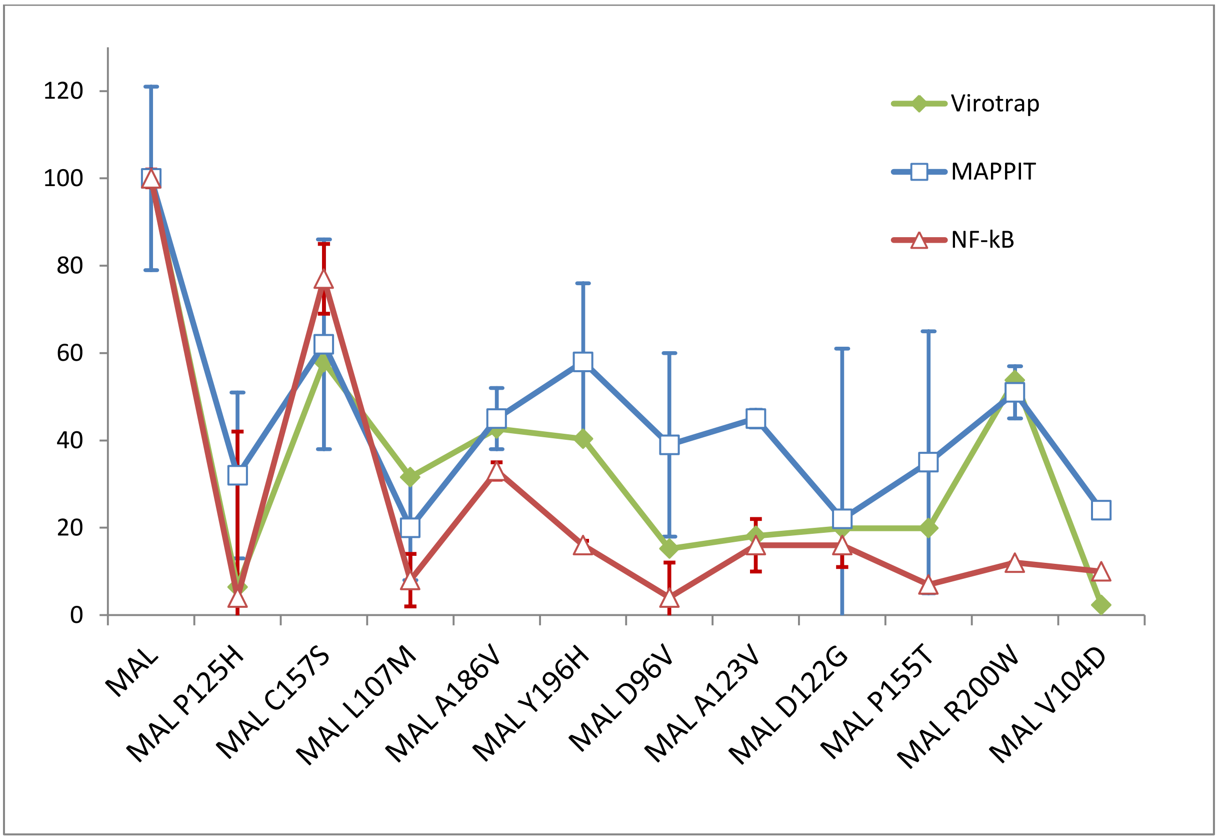

Supplementary Fig. 4: Quantification of the interaction between MYD88 TIR and MAL mutant proteins. Binary Virotrap assays for the interaction between the TIR domain of MYD88 and MAL variants. Enriched particles were lysed in loading buffer and prey protein levels were quantified using gel imaging software (Li-Cor, ODYSSEY®) after separation by SDS-PAGE. The quantity of mutant MAL proteins in the particles was plotted as a percentage of the interaction between MYD88 TIR and the wild type MAL protein (100%). The values for the MAPPIT assay and NF-κB signaling were obtained from Bovijn and colleagues[2]. The Western analysis in **Fig. 2b** was performed as 3 independent biological repeats. A representative experiment out of 3 biological repeats is shown in Fig. 2c (used for the quantification presented here).

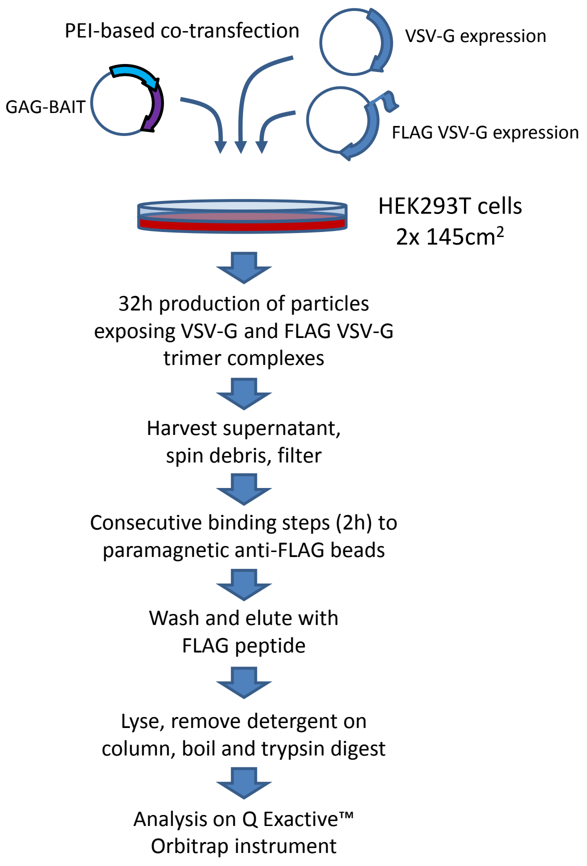

Supplementary Fig. 5: Schematic overview of the single step purification Virotrap protocol. Cells are co-transfected with plasmids encoding a GAG-bait construct and a 1/3 ratio of plasmids encoding untagged VSV-G vs 2/3 FLAG-tagged (or E-tagged) VSV-G protein. After harvest of supernatant and filtering, the particles are captured on paramagnetic beads, specifically eluted by the FLAG peptide and lysed. After detergent removal, boiling and trypsin digestion, the samples are analyzed by mass spectrometry. More details are described in the Materials and Methods section of the main article. A general outline of a Virotrap experiment is described in the Supplementary notes section.

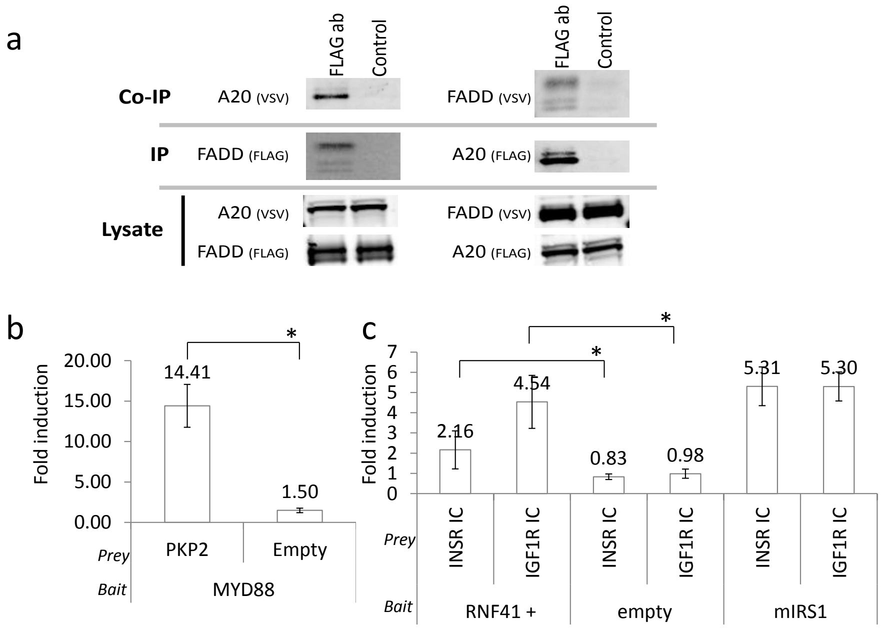

Supplementary Fig. 6: Confirmation of novel interactions detected with Virotrap. a. Confirmation of the interaction between FADD and A20. Co-immunoprecipitation experiments showed specific binding of A20 to immune-precipitated FADD (left panels), or of FADD to immune-precipitated A20 (right panels). Tagged proteins (FLAG and VSV tags) were expressed in HEK293T cells and were precipitated after lysis using paramagnetic anti-tag beads or control beads. The co-precipitated proteins were revealed by anti-VSV antibodies. A representative experiment out of 2 biological repeats is shown. Uncropped gel images and molecular weight markers are presented in **Supplementary Fig. 10**. **b**. **MAPPIT confirmation of the interaction between MYD88 and plakophilin 2 (PKP2)**. MYD88 TIR was fused as a bait protein to the MAPPIT bait vector (pCLG). Empty prey control vector was compared to a prey vector expressing PKP2. Luciferase activity was measured 4 times (technical replicates). One out of two independent biological experiments is shown. **c**. **MAPPIT confirmation for the interaction of RNF41 with the intracellular domain of the insulin receptor (INSR IC) and the insulin-like growth factor receptor 1 (IGF1R IC)**. Murine Insulin Receptor Substrate 1 (mIRS1) as bait was used as a positive control, while an empty bait vector was used as negative control. The intracellular parts of the insulin and insulin-like growth factor receptors were fused to the MAPPIT prey construct (pMG1). Luciferase activity was measured 4 times (technical replicates). One out of two independent biological experiments is shown.

We used the paired two-sample t-test to derive P values for both **b** and **c**. The error bars on the average value in plots **b** and **c** show the standard deviation.

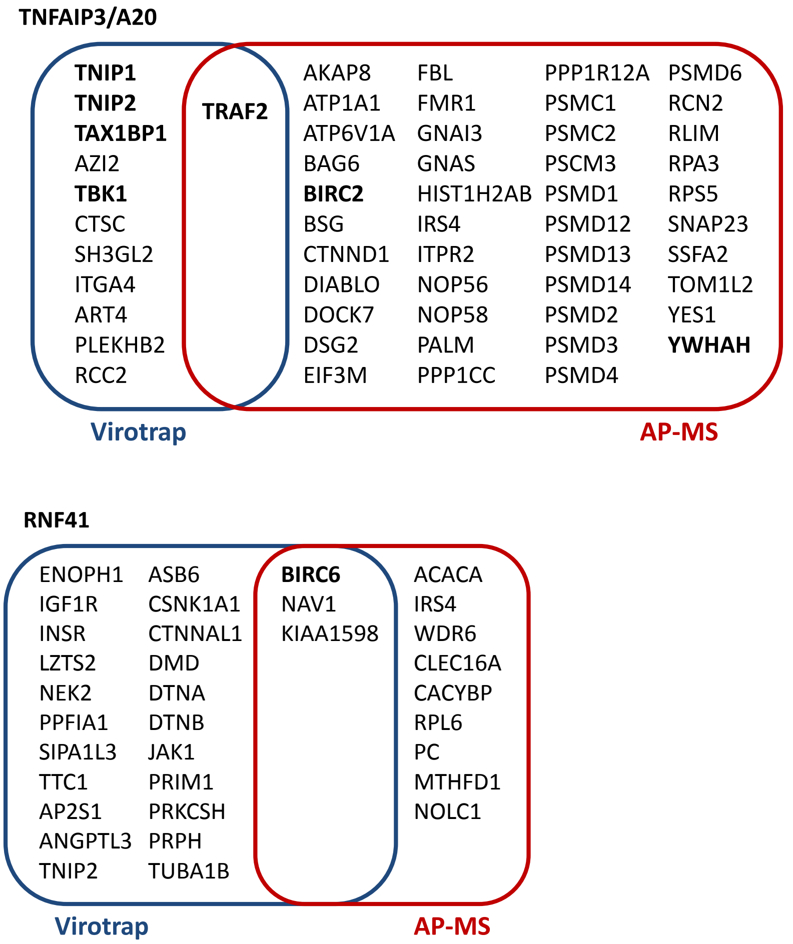

Supplementary Fig. 7. When compared to AP-MS, Virotrap reveals a complementary interactome. TNFAIP3 (A20) and RNF41 were analyzed by classical FLAG-based AP-MS and Virotrap. The FLAG-tagged (AP-MS) and GAG-fusion (Virotrap) bait proteins were expressed from the same vector in HEK293T cells. For AP-MS, we used the protocol from Kean *et al.*[3] and analyzed the data using SAINT[4] on 3 biological repeats combined with 2 EGFP AP-MS control experiments and 8 relevant CRAPOME controls (www.crapome.org)[5]. Proteins with a SAINT score above 0.8 are reported. For TNFAIP3, we additionally removed about 30 cytoskeletal proteins (based on GO annotation) from the AP-MS data, which are likely background proteins. For Virotrap, we used 19 control experiments and removed all these protein identifications (including single peptide identifications) from the list of proteins identified in a triplicate experiment. Only proteins with two or more identified peptides were retained. Proteins in bold were annotated as interaction partners in BioGRID 3.2. An overview of the AP-MS experiments can be found in **Supplementary Table 4** below. Files containing protein spectral counts and peptide to spectra matches counts are provided as additional information.

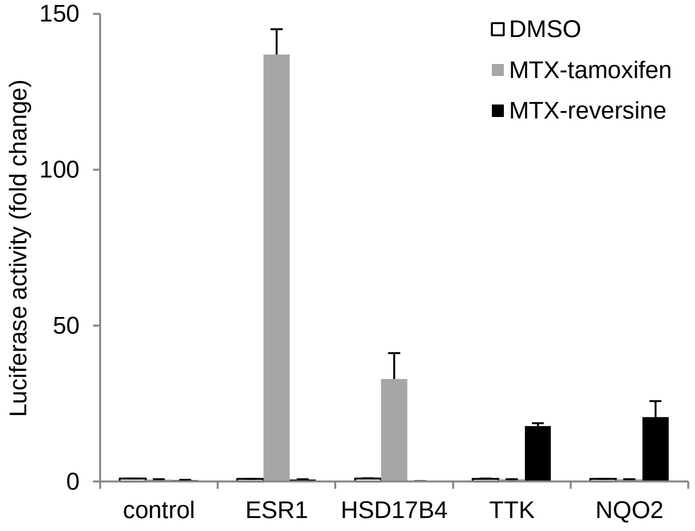

Supplementary Fig. 8: MASPIT[6] confirmation of novel protein targets for tamoxifen and reversine. HEK293T cells were transfected with the eDHFR bait construct and different prey constructs (empty prey construct, ESR1, HSD17B4, TTK and NQO2). After transfection, cells were treated with DMSO as control, 1 μM methotrexate-tamoxifen (MTX-tamoxifen) or 1 μM methotrexate-reversine (MTX-reversine). Estrogen receptor 1 (ESR1) served as positive control for tamoxifen while TTK is a known target for reversine. The plots show the average fold changes in luciferase activity of cells treated with bivalent molecules or DMSO versus untreated cells. Experiments were performed as biological triplicates, with repeat luciferase measurements (4x) of parallel transections.

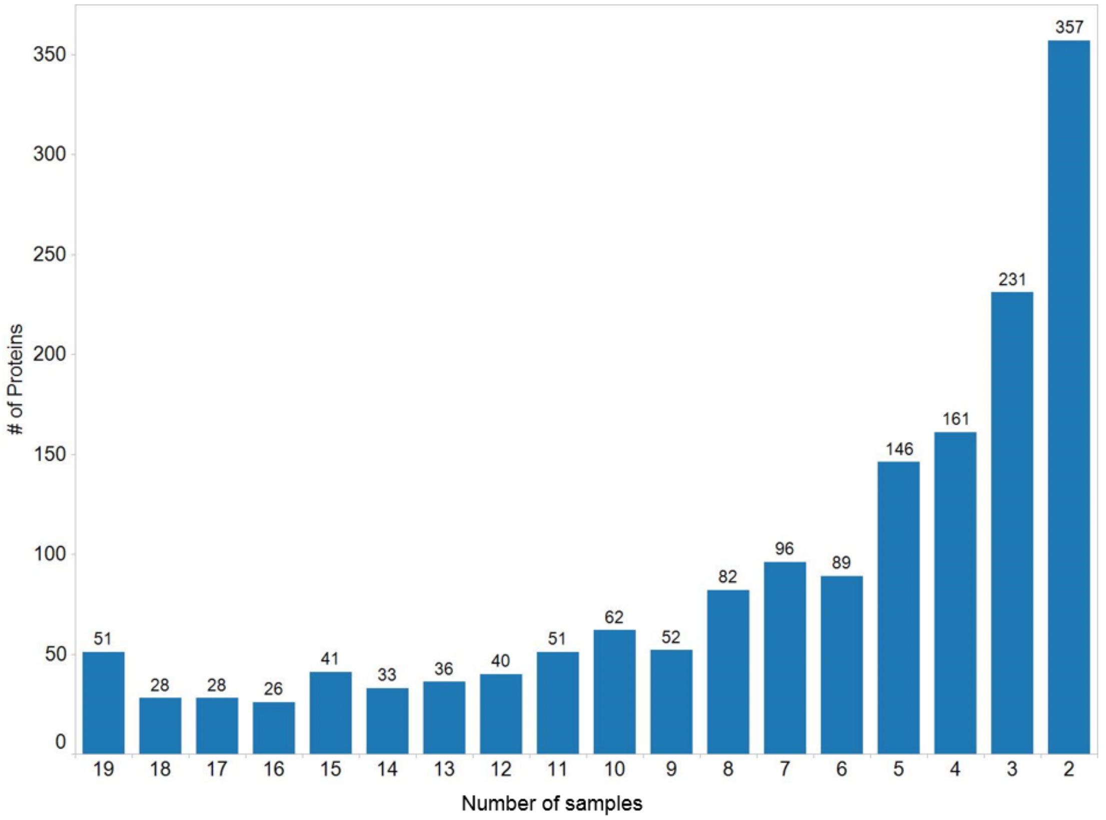

Supplementary. Fig. 9a: Number of recurrent proteins in function of the number of samples in Virotrap. Bars in the graph show the number of proteins identified in the total number of samples out of 19 control Virotrap experiments (e.g. 41 proteins were found recurrently in 15 out of 19 control samples). This analysis includes all human and bovine (Foetal Calf Serum) proteins, and proteins required for the system (GAG and VSV-G/FLAG - VSV-G). 174 Proteins are highly recurrent in these 19 Virotrap reference samples (identified in at least 15 of the samples, i.e. combined bars from 19, 18, 17, 16 and 15 samples).

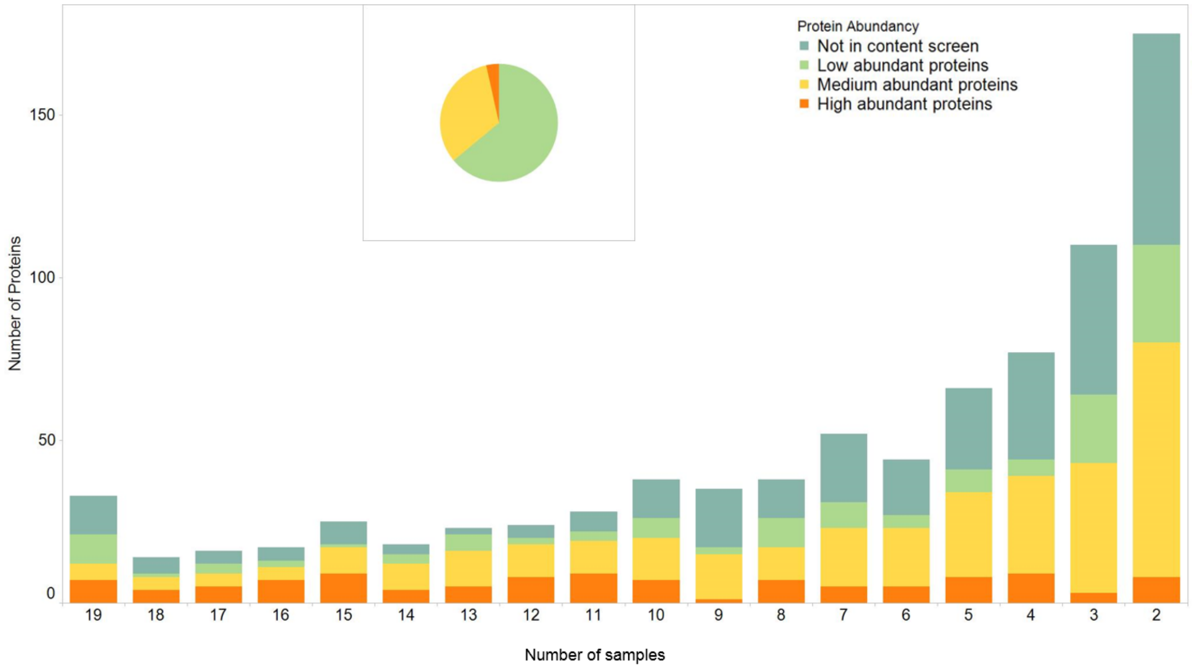

Supplementary Fig. 9b: Recurrence of human proteins in 19 control Virotrap samples with distribution of abundance in HEK293T cells. Abundance was defined by using spectral counts obtained from a comprehensive shotgun analysis on a HEK293T cell lysate. Low abundant proteins were defined as identified proteins with less than 10 spectral counts, medium abundant proteins were identified with spectral counts between 10 and 100. High abundant proteins have more than 100 spectral counts. The pie chart insert shows the distribution of these abundance ranges for the whole HEK293T proteome as a reference. Only human proteins were assessed here (bovine serum proteins and HIV proteins were removed from this analysis).

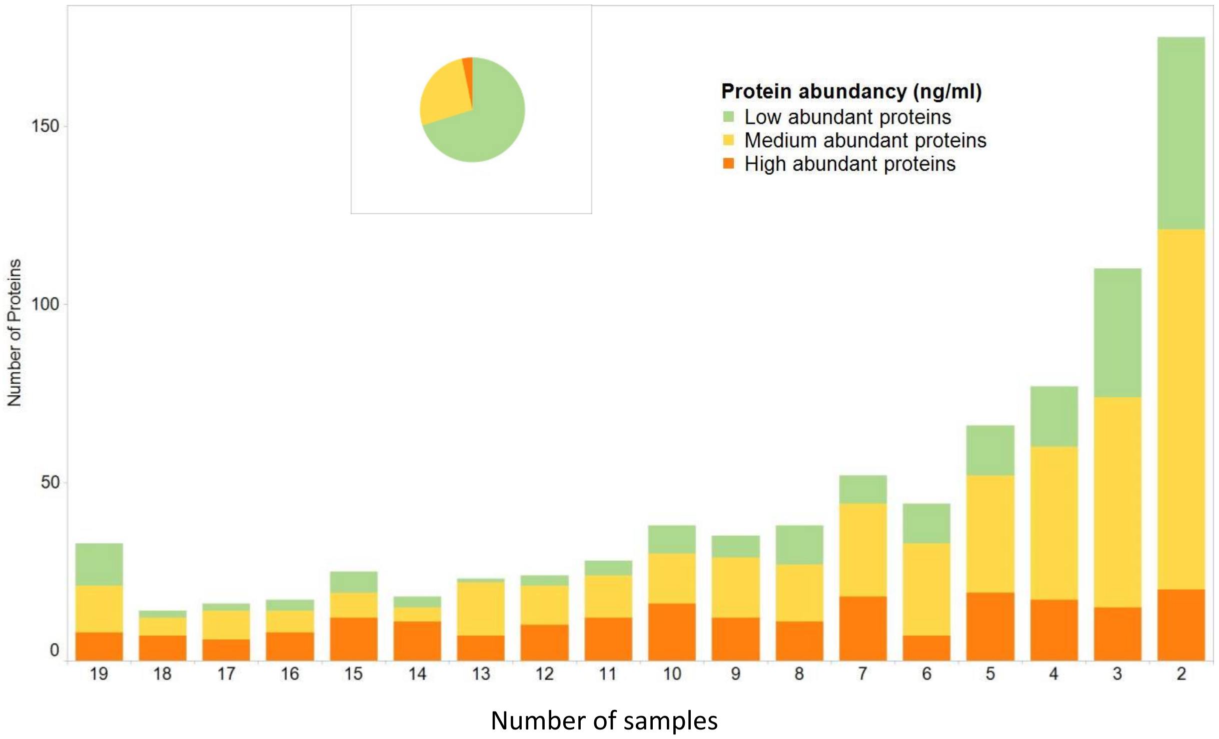

Supplementary Fig. 9c: Abundance distribution (based on MOPED dataset) vs. recurrence for human proteins in 19 Virotrap reference samples. Same as **Supplementary Fig. 9b**. but with the MOPED database[7] as a source for protein concentrations. In this case, low abundant proteins were defined as having a concentration below 5 µg/ml, medium abundant proteins range between 5 and 100 µg/ml, while high abundant proteins have concentrations over 100 µg/ml. The pie chart insert shows the distribution of these concentration ranges in the MOPED dataset for HEK293 cells.

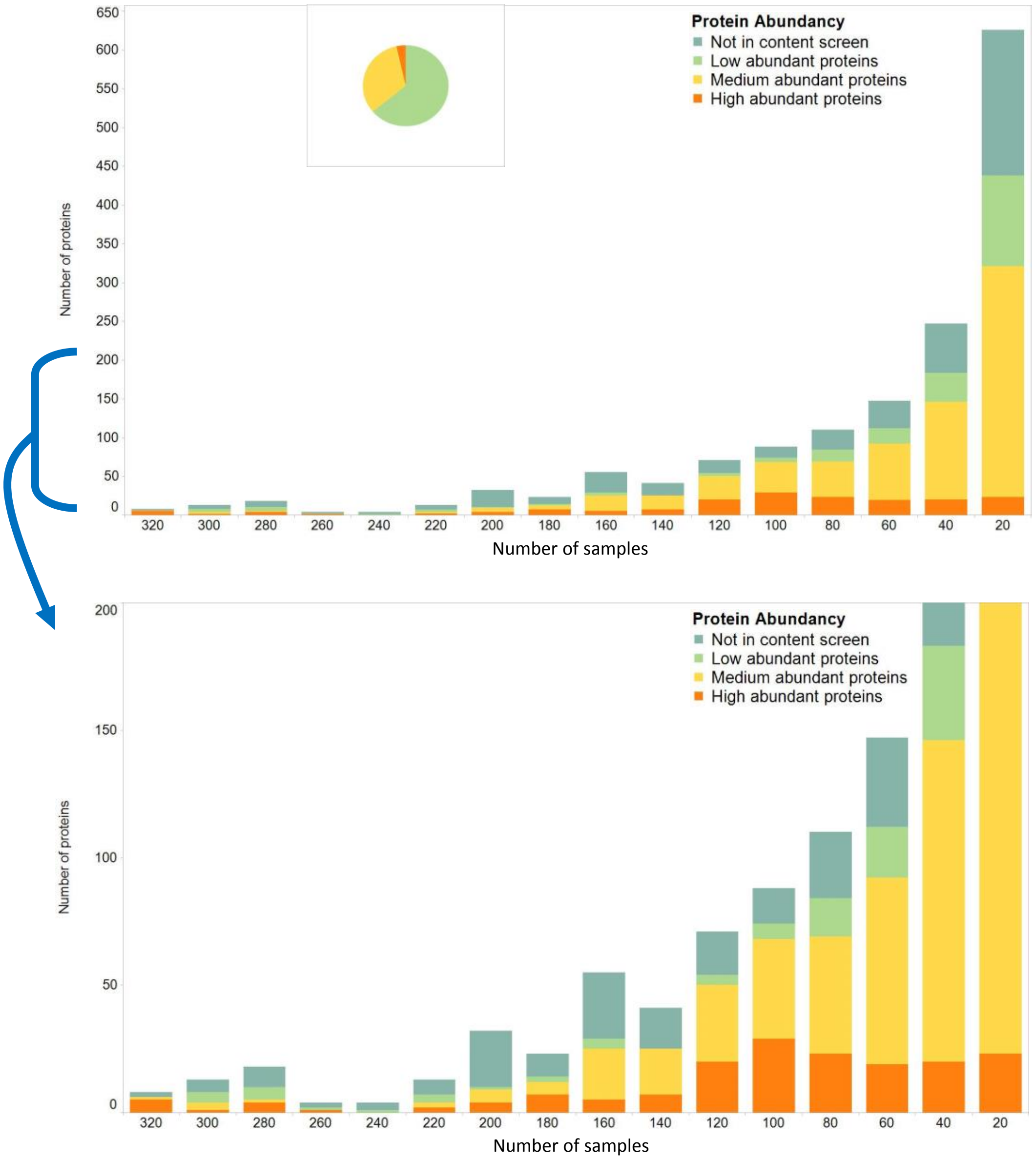

Supplementary Fig. 9d: Abundance distribution in the full human CRAPOME (v1.0.) dataset. Distribution of low, medium and high abundant proteins in function of the recurrence in 343 samples in the CRAPOME database (V1.0 *Homo sapiens,*[5]). As in 9b., the HEK293T shotgun proteome was used as a reference to assess protein abundance (with reference distribution in the HEK293T proteome shown in the pie chart insert). The lower panel zooms in on the region up to 200 proteins.

Non-cropped gel images

Fig 2a

1	GAG-GRAP2 (**I**)	E-LCP2 (**A**)
2	GAG (**M**)	E-LCP2
3	GAG-control (CKS1B)	E-LCP2
4	GAG-LCP2 (**J**)	E-GRAP2 (**B**)
5	GAG	E-GRAP2
6	GAG-control (CKS1B)	E-GRAP2
7	GAG-CKS1B (**K**)	E-CDK2 (**C**)
8	GAG	E-CDK2
9	GAG-control (LCP2)	E-CDK2
10	GAG-CDK2 (**L**)	E-CKS1B (**D**)
11	GAG	E-CKS1B
12	GAG-control (EGFP;**N**)	E-CKS1B
13	GAG-S100B (**G**)	E-S100A1 (E)
14	GAG	E-S100A1
15	GAG-control (EGFP)	E-S100A1
16	GAG-S100A1 (**H**)	E-S100B (F)
17	GAG	E-S100B
18	GAG-control (EGFP)	E-S100B

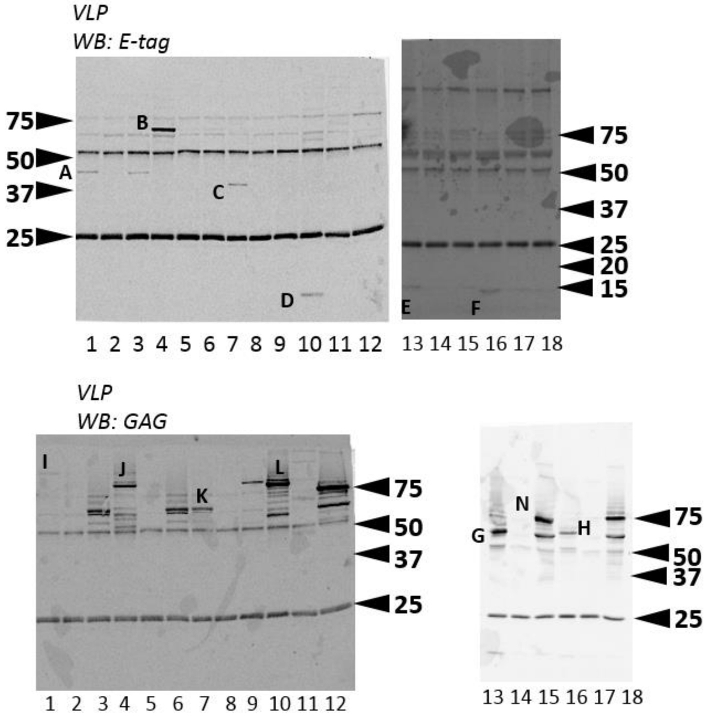

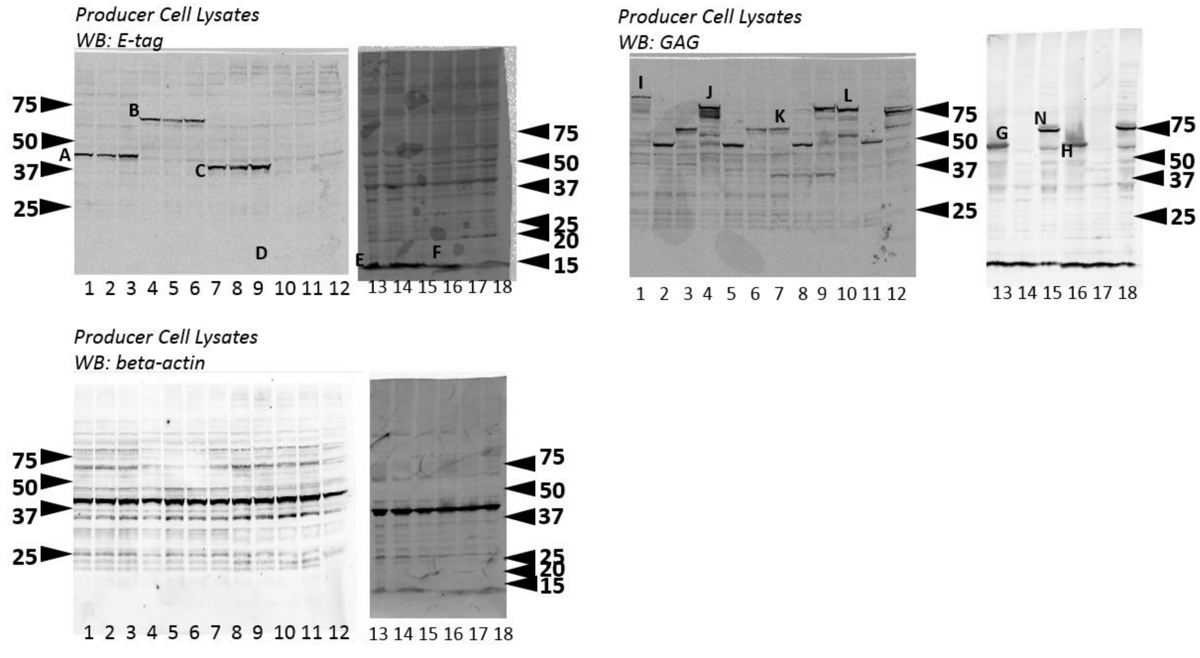

Fig. 2b

1	GAG-MYD88 TIR (**C**)	-
2	GAG-MYD88 TIR	MYC-FADD (**A**)
3	GAG-MYD88 TIR	MYC-MAL (**B**)
4	GAG-MYD88 TIR	MYC-MAL P125H
5	GAG-MYD88 TIR	MYC-MAL C157S
6	GAG-MYD88 TIR	MYC-MAL L107M
7	GAG-MYD88 TIR	MYC-MAL A186V
8	GAG-MYD88 TIR	MYC-MAL Y196H
9	GAG-MYD88 TIR	MYC-MAL D96V
10	GAG-MYD88 TIR	MYC-MAL A123V
11	GAG-MYD88 TIR	MYC-MAL D122G
12	GAG-MYD88 TIR	MYC-MAL P155T
13	GAG-MYD88 TIR	MYC-MAL R200W
14	GAG-MYD88 TIR	MYC-MAL V104D
15	GAG-EGFP (**D**)	MYC-MAL
16	GAG-EGFP	MYC-FADD

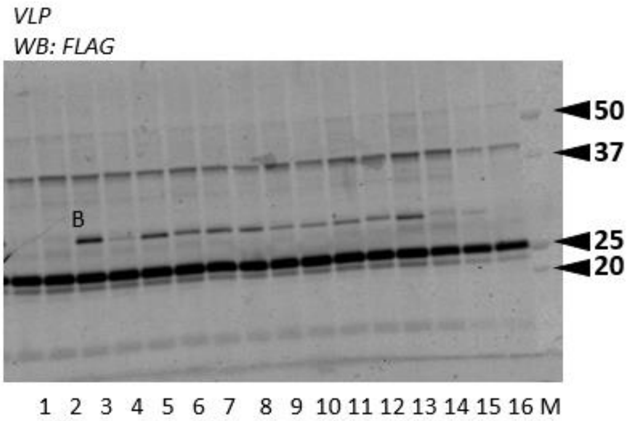

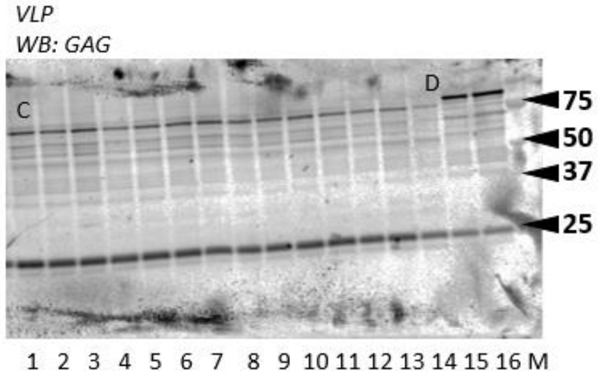

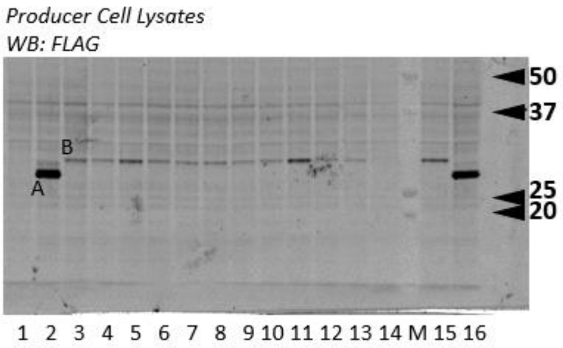

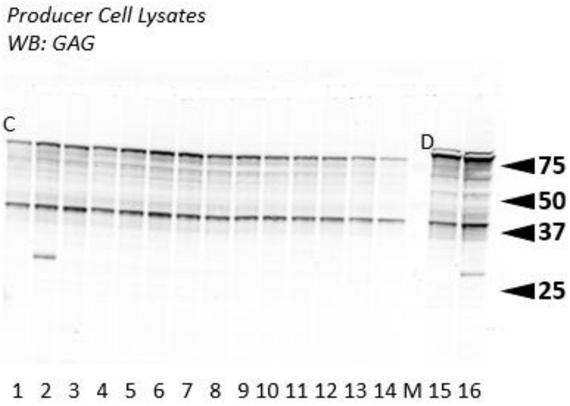

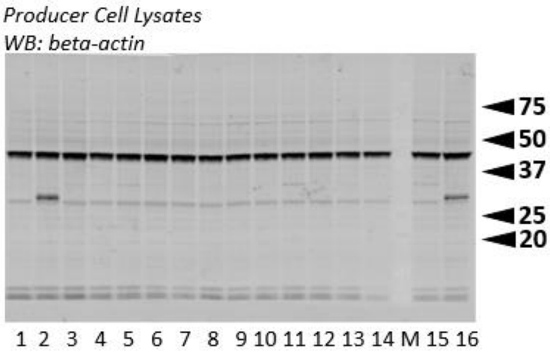

Supplementary Figure 1a

1	GAG-RAS (**C**)	FLAG-control (IRS4 SH2; **A**)
2	GAG-RAS	FLAG-RAF (**B**)
3	GAG-control (EGFP; **D**)	FLAG-RAF
4	-	FLAG-RAF

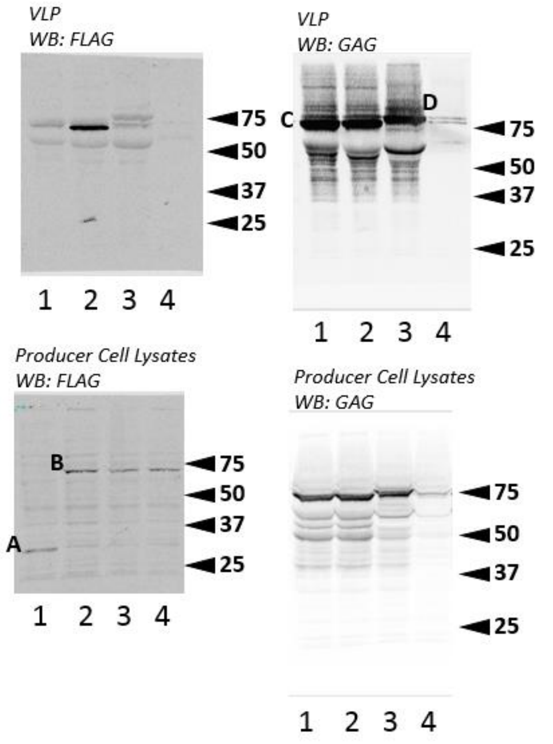

Supplementary Figure 1b

1	GAG-RAS (**C**)	FLAG-control (IRS4 SH2; **A**)
2	GAG-RAS	FLAG-RAF (**B**)
3	GAG-control (EGFP; **D**)	FLAG-RAF
4	-	FLAG-RAF

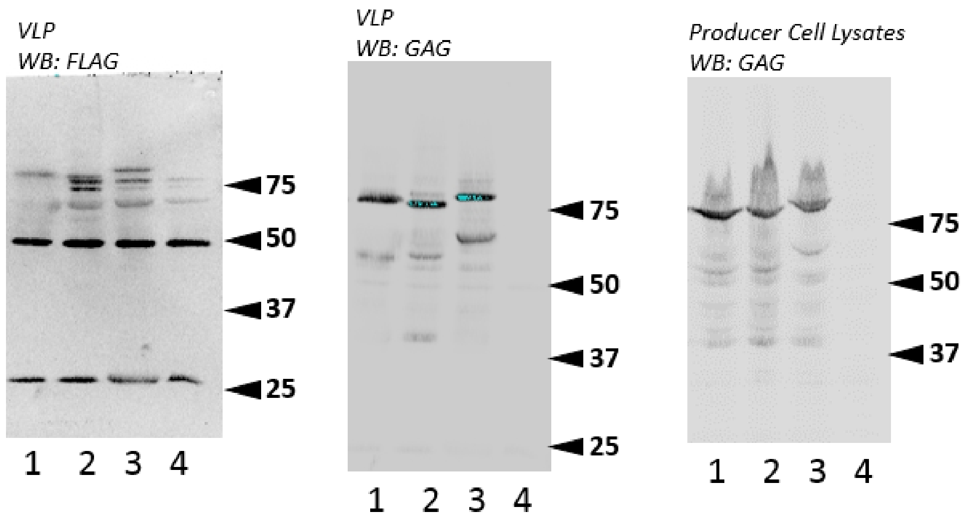

Supplementary Figure 6a

1 FLAG-FADD VSV-A20

2 FLAG-A20 VSV-FADD

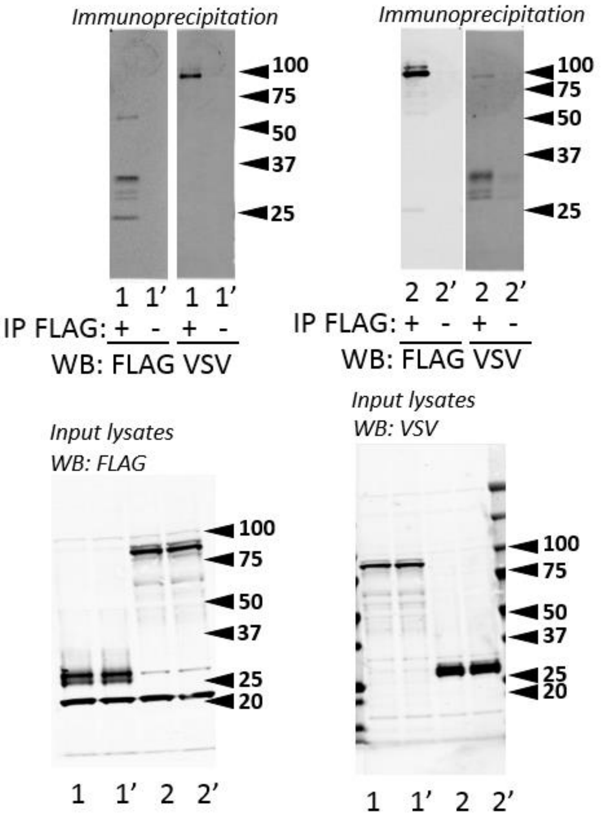

Supplementary Fig. 10: Full image views of the PAGE gels shown in this manuscript.

Supplementary Table 1: Overview of the different Virotrap runs performed in this study. The number of spectra, identified spectra or peptide to spectra matches (PSM), different peptides (sequences) and proteins are shown. The False Discovery Rate (FDR) for identification was determined by searches against a reversed version of the Virotrap database (containing human, bovine, HIV, EGFP, VSV-G and FLAG-VSV-G sequences). Protein spectral count and peptide spectral count files are provided separately as excel files.

Sample	Spectra	PSM	Sequences	Proteins	FDR
Control 1	7464	1502	1197	410	0.40
Control 2	8894	2343	799	336	1.15
Control 3	6654	1968	1317	311	0.30
Control 4	6368	1774	1285	345	0.45
Control 5	12593	3363	2869	837	0.06
Control 6	10613	2241	1970	643	0.00
Control 7	14787	2044	1373	422	0.00
Control 8	14822	2604	2146	596	0.23
Control 9	28258	8280	2989	903	0.05
Control 10	14501	3427	2879	819	0.03
Control 11	14632	3907	3378	973	0.08
Control 12	14267	3872	3286	942	0.05
Control 13	14851	3561	2901	791	0.08
Control 14	18240	1543	1157	447	0.26
Control 15	16397	1929	1538	615	0.00
Control 16	15455	1733	1407	552	0.06
Control 17	15014	1902	1415	571	0.00
Control 18	15139	1469	1022	396	0.00
Control 19/CDK2 1	14682	1706	1264	503	0.12
CDK2 2	13902	3701	3059	858	0.14
CDK2 3	15544	3415	2733	806	0.09
FADD 1	8277	2926	1798	539	1.03
FADD 2	7123	2554	1537	421	0.20
FADD 3	6391	1744	1264	318	0.52
FADD 4	7010	2317	1435	418	0.52
MYD88 1	16240	4380	3714	1036	0.00
MYD88 2	13534	4800	4151	1285	0.06
MYD88 3	13482	4286	3673	1074	0.07
NEMO 1	12816	2177	1333	371	0.18
NEMO 2	13170	2100	1776	540	0.05
NEMO 3	14989	4235	3119	875	0.05
NEMO 4	14476	4671	3229	942	0.04
RNF41 1	12974	3346	2914	833	0.09
RNF41 2	14625	1764	1266	402	0.11
RNF41 3	16934	2298	1835	567	0.04
TANK 1	14500	1942	1557	506	0.05
TANK 2	14790	4816	3995	1169	0.02

TANK 3	14876	3924	3306	1025	0.03
A20 1	12110	2823	2396	688	0.00
A20 2	13808	3726	3055	767	0.27
A20 3	14109	4103	3385	855	0.22
A20 + TNF 1	15668	2066	1724	519	0.19
A20 + TNF 2	17314	2728	1953	589	0.22
A20 + TNF 3	17065	2621	1970	604	0.19
A20 + TNF 4	16872	2072	1577	495	0.39
A20 + TNF 5	12909	2017	1529	479	0.35
DMSO 1	14695	3736	2512	807	0.03
DMSO 2	14049	3542	3029	842	0.09
DMSO 3	16890	2217	1705	566	0.18
DMSO 4	16390	2171	1785	601	0.05
Simvastatin 1	14390	3548	2534	833	0.00
Simvastatin 2	17267	1606	1025	338	0.00
Simvastatin 3	19726	2821	1979	590	0.21
Tamoxifen 1	13342	2627	2372	752	0.11
Tamoxifen 2	15213	1496	1192	436	0.07
Tamoxifen 3	17750	2958	1955	612	0.03
Reversine 1	13891	3102	2478	800	0.03
Reversine 2	16582	2249	1876	624	0.04
Reversine 3	13282	2481	2205	611	0.20
Reversine 4	12701	2664	2381	658	0.18

Supplementary Table 2: Overview of the different AP-MS experiments performed in this study. The number of spectra, identified spectra or peptide to spectra matches (PSM), peptides (sequences) and proteins are shown.

Sample	Spectra	PSM	Sequences	Proteins
AP-MS A20 1	17444	2191	1465	488
AP-MS A20 2	15665	3055	2475	969
AP-MS A20 3	15980	3874	3154	1161
AP-MS EGFP 1	19558	1964	1729	586
AP-MS EGFP 2	17892	1432	1244	539
AP-MS RNF41 1	15839	1965	1500	419
AP-MS RNF41 2	16652	1904	1346	363
AP-MS RNF41 3	20817	3581	2382	706

Supplementary note 1: Lay-out of a Virotrap experiment.

Design

As with other co-complex approaches such as AP-MS, a well-conceived design is essential to perform successful Virotrap experiments. In this manuscript, we use a classical and intuitive 'black list strategy' implying that a number of control experiments should be performed in parallel to allow removal of all proteins detected in these control experiments from the real experiment(s). We used 19 control experiments with different GAG-bait constructs. These baits consisted of EGFP or unrelated bait proteins. It should be clear that the selection of unrelated bait proteins is critical to prevent false negatives by removing proteins that are in the same complex or 'network'. In an ideal setting, three replicate experiments for the bait should be performed. **Fig. 3** and **Fig. 4** show the design of the experiments and the number of replicates that were performed for the studies in this report.

Experimental

Virotrap experiments are then processed as described in **Supplementary Fig. 5** and in the materials and methods section associated to this report. Briefly, cells are seeded on day one in 2 T175 tissue culture flasks for each replicate and control experiment. Sub-confluent cell cultures are then transfected the next day with GAG-bait plasmid and plasmids that express VSV-G and FLAG-tagged VSV-G to allow particle purification. Medium is replaced and cells are left to produce Virotrap particles for 32 hours. Different treatments can be applied during the production process (e.g. TNFalpha and MTX-PEG6-simvastatin). The harvested supernatant is then centrifuged and filtered to remove cellular debris. Magnetic Anti-FLAG beads are added to the supernatant and particles are allowed to bind. After binding, particles are washed once before they are released by the FLAG peptide. After SDS lysis, the samples are processed using detergent removal columns, boiled and digested by trypsin. After acidification, samples are analyzed by mass spectrometry.

Analysis

MS spectra were searched against a human database, complemented with bovine (for serum proteins associated to particles) and HIV proteins. Sequences for VSV-G, FLAG-VSV-G and EGFP were added as well. A full list of all identified proteins, including proteins identified with a single peptide, is generated for the bait control experiments. The list obtained for the three replicates from the bait of interest is challenged against the control list to eliminate all proteins that overlap. Recurrence in the three repeat experiments of proteins identified with at least two peptides is a good way for prioritization of candidates.

Alternative analysis options

Different tools for filtering co-complex data are currently available[8]. We were unable to define good parameter settings for SAINT analysis [4], a well-known and powerful tool for AP-MS analysis. Processing the data with SFINX[9] provides high retrieval of known interaction partners as demonstrated for TTK binding to reversine. Preliminary results suggest that a label-free quantification approach using a MAXQUANT/PERSEUS [10 11] workflow can be applied as well. Although less control samples are required, this strategy implies rigorous protocol routine and combined processing of both control and test samples. MS analysis should be performed under highly similar

conditions (e.g. same column, consecutive runs,…). When well performed, such LFQ analysis allows sensitive detection of subtle interaction differences with reduced risk of eliminating true interactions (reduction of false negatives).

Supplementary note 2: Virotrap background analysis

An important issue in typical MS co-complex strategies relates to the removal of non-specific background proteins [5]. In this study we defined background proteins very stringently as proteins that were identified in Virotrap experiments that are unrelated to the actual experiment (see also previous section). A distribution of the occurrence of background proteins in 19 Virotrap control samples is shown in **Supplementary Fig. 9a**. It is clear that about 174 proteins occur in virtually all samples (identified in 15/19 samples). As expected, GAG and VSV-G are found in all samples. Other recurrent contaminants are serum proteins (e.g. A2M, albumin) and host proteins involved in sorting and budding of virus-like particles (e.g. VPS37B, MVB12A) as well as structural proteins (e.g. ACTB, EZR). Further, known interactors of GAG are found in most of the samples (Cyclophilin/FKBPA, ALIX/PDCD6IP, TSG101 and CNP). Note that the interaction between Cyclophilin and GAG is very weak ($K_d = 1.6 \times 10^{-5}$ M [12]) supporting the notion that Virotrap can trap weak interactions. To assess the effect of protein abundance in the Virotrap background, we looked at the recurrence of human proteins in the 19 reference samples. We used data from a comprehensive shotgun analysis on the host cells to obtain a protein content list with spectral counting as a measure for protein abundance (**Supplementary Fig. 9b**). In addition, we have also used protein concentration data for HEK293 cells obtained from the MOPED database[7] (**Supplementary. Fig. 9c**). Compared to the abundance distribution in HEK293T cells, we observed a relative increase in high abundant proteins in the Virotrap samples, which is largely comparable to what is observed in the CRAPOME (**Supplementary Fig. 9d**, v1.0; 343 experiments[5]). Virotrap however showed a high recurrence of proteins with lower abundance, reflecting the unique biological mechanisms underlying the method.

Supplementary References

1. Braun P*, et al.* An experimentally derived confidence score for binary protein-protein interactions. *Nature methods* **6**, 91-97 (2009).

2. Bovijn C, Desmet AS, Uyttendaele I, Van Acker T, Tavernier J, Peelman F. Identification of binding sites for myeloid differentiation primary response gene 88 (MyD88) and Toll-like receptor 4 in MyD88 adapter-like (Mal). *The Journal of biological chemistry* **288**, 12054-12066 (2013).

3. Kean MJ, Couzens AL, Gingras AC. Mass spectrometry approaches to study mammalian kinase and phosphatase associated proteins. *Methods (San Diego, Calif)* **57**, 400-408 (2012).

4. Choi H*, et al.* SAINT: probabilistic scoring of affinity purification-mass spectrometry data. *Nature methods* **8**, 70-73 (2011).

5. Mellacheruvu D*, et al.* The CRAPome: a contaminant repository for affinity purification-mass spectrometry data. *Nature methods* **10**, 730-736 (2013).

6. Caligiuri M*, et al.* MASPIT: three-hybrid trap for quantitative proteome fingerprinting of small molecule-protein interactions in mammalian cells. *Chemistry & biology* **13**, 711-722 (2006).

7. Kolker E*, et al.* MOPED: Model Organism Protein Expression Database. *Nucleic acids research* **40**, D1093-1099 (2012).

8. Meysman P*, et al.* Protein complex analysis: From raw protein lists to protein interaction networks. LID - 10.1002/mas.21485 [doi]. *Mass Spectrom Rev*, (2015).

9. Titeca K*, et al.* SFINX: Straightforward Filtering Index for Affinity Purification-Mass. *J Proteome Res* **15**, 332-338 LID - 310.1021/acs.jproteome.1025b00666 [doi] (2016).

10. Cox J, Mann M. MaxQuant enables high peptide identification rates, individualized p.p.b.-range. *Nature biotechnology* **26**, 1367-1372 LID - 1310.1038/nbt.1511 [doi] (2008).

11. Tyanova S, Temu T, Carlson A, Sinitcyn P, Mann M, Cox J. Visualization of LC-MS/MS proteomics data in MaxQuant. *Proteomics* **15**, 1453-1456 (2015).

12. Yoo S, Myszka DG, Yeh C, McMurray M, Hill CP, Sundquist WI. Molecular recognition in the HIV-1 capsid/cyclophilin A complex. *Journal of molecular biology* **269**, 780-795 (1997).
